# Enzymes and membrane proteins of ADSOL-preserved red blood cells

**DOI:** 10.1590/S1516-31802000000200003

**Published:** 2000-03-02

**Authors:** Maria Sueli Soares Leonart, Aguinaldo José Nascimento, Kimiyo Nonoyama, Cinthia Barbosa Pelissari, Orlando Cesar de Oliveira Barretto

**Keywords:** Red cell ageing, Red cell membrane proteins, Red cell enzymes, Red cell preservation, ADSOL

## Abstract

**CONTEXT:**

The preservative solution ADSOL (adenine, dextrose, sorbitol, sodium chloride and mannitol) maintains red cell viability for blood transfusion for 6 weeks. It would be useful to know about its preservation qualities over longer periods.

**OBJECTIVE:**

To determine some red cell biochemical parameters for periods of up to 14 weeks in order to determine whether the red cell metabolism integrity would justify further studies aiming at increasing red cell preservation and viability.

**DESIGN:**

Biochemical evaluation designed to study red cell preservation.

**SETTING:**

São Paulo University erythrocyte metabolism referral center.

**SAMPLE:**

Six normal blood donors from the University Hospital of the Universidade Federal do Paraná, Curitiba, Brazil.

**MAIN MEASUREMENTS:**

Weekly assay of erythrocyte adenosine-5′-triphosphate (ATP), 2,3-diphosphoglycerate (2,3DPG), hexokinase (HX), phosphofructokinase (PFK), pyruvate kinase (PK), glucose-6-phosphate dehydrogenase (G-6-PD), 6-phosphogluconic dehydrogenase (6-PGD), glyceraldehyde-3-phosphate dehydrogenase (GAPD), glutathione reductase (GR), glutathione peroxidase (GSHPx), plasma sodium and potassium, blood pH, and membrane proteins of red cells preserved in ADSOL were studied during storage for 14 weeks storage.

**RESULTS:**

During ADSOL preservation, erythrocyte ATP concentration decreased 60% after 5 weeks, and 90% after 10 weeks; the pH fell from 6.8 to 6.4 by the 14th week. 2,3-DPG concentration was stable during the first week, but fell 90% after 3 weeks and was exhausted after 5 weeks. By the end of the 5th week, an activity decrease of 16-30% for Hx, GAPD, GR, G-6-PD and 6-PGD, 35% for PFK and GSHPx, and 45% for PK were observed. Thereafter, a uniform 10% decay was observed for all enzymes up to the 14th week. The red blood cell membrane proteins did not show significant alterations in polyacrylamide gel electrophoresis (SDS-PAGE) during the 14 weeks.

**CONCLUSION:**

Althoughthe blood viability was shown to be poor from the 6th week up to the 14th week of storage due to ATP and 2,3-DPG depletion, the other biochemical parameters remained in fairly good condition for longer storage. As there is a gradual and uniform decay in activity throughout these 14 weeks, it seems that ADSOL-preserved red cells may be used as red cell enzyme standards and membrane proteins as well.

## resumo

**CONTEXTO:**

A solução preservadora ADSOL (adenina, dextrose, sorbitol, cloreto de sódio e manitol) mantém a viabilidade dos glóbulos vermelhos para transfusão durante seis semanas. Seria assim útil determinar sua preservação por tempos maiores.

**OBJETIVO:**

Determinar alguns parâmetros bioquímicos eritrocitários até 14 semanas visando saber se a integridade do metabolismo eritrocitário justificaria estudos posteriores com o propósito de alongar sua preservação e viabilidade.

**TIPO DE ESTUDO:**

Avaliação bioquímica para avaliar a preservação de hemácias.

**LOCAL:**

Centro de referência de metabolismo eritrocitário da Faculdade de Medicina da USP, São Paulo e Universidade Federal do Paraná, Curitiba.

**AMOSTRA:**

Seis doadores de sangue do hospital universitário da Universidade Federal do Paraná, Curitiba, Brasil.

**VARIÁVEIS ESTUDADAS:**

Foi realizada a determinação semanal de adenosina-5′-trifosfato, 2,3-difosfoglicerato, hexoquinase, fosfofrutoquinase, piruvato quinase, glicose-6-fosfato desidrogenase, 6-fosfogliconico desidrogenase, gliceraldeido-3-fosfato desidrogenase, glutationa redutase, glutationa peroxidase, bem como a dosagem de sódio e potássio plasmáticos, pH sangüíneo, e a determinação das proteínas da membrana eritrocitária por eletroforese em gel de poliacrilamida.

**RESULTADOS:**

Durante a preservação o ATP caiu 60% em cinco semanas, e 90% depois de 10 semanas. O 2,3-DPG permaneceu estável durante a primeira semana, caiu 90% depois de três semanas e se exauriu depois de cinco semanas. O pH decresceu de 6,8 na primeira semana a 6,4 na 14a semana. Depois de cinco semanas houve diminuição de 16 a 31% das atividades da hexoquinase, gliceraldeído-3-fosfato desidrogenase, glutationa redutase, e 45% da piruvato quinase. Em seguida, observou-se um decréscimo de 10% para todas enzimas até a 14ª semana. A eletroforese em gel de poliacrilamida das proteínas da membrana eritrocitária não revelou alterações nas concentrações relativas das bandas durante e ao cabo das 14 semanas.

**CONCLUSÕES:**

Embora a viabilidade do sangue seja pobre da 6ª à 14^a^ semana, devido à depleção de ATP e de 2,3-DPG, os demais parâmetros bioquímicos decaíram gradualmente. Este achado pode sugerir que os glóbulos vermelhos preservados em ADSOL possam ser utilizados como padrões de enzimas eritrocitárias e de proteínas da membrana.

## INTRODUCTION

Red cell preservative solutions have been designed over the last two decades^1-7^ with the aim of increasing blood viability for transfusion. ADSOL^2^ preservative solution, composed of adenine, dextrose, sodium chloride and mannitol, has been employed worldwide. Red cells maintained in ADSOL are viable for transfusion for up to 7 weeks.^7,8^

Studies on red cell preservation have aimed at keeping ATP and 2,3-DPG concentrations for as long as possible, and many researchers have been able to maintain them for up to 7 weeks.^7,8^ Although these remarkable results have been achieved by the addition of several compounds like adenine, inosine, sorbitol, ascorbic acid, etc., they could only be obtained because the red cell metabolism was still working well, as the glycolytic and pentose shunt enzymes and also the membrane proteins keep fairly good activity and integrity levels. Consequently, glycolytic kinases, glycolytic and non-glycolytic dehydrogenases and membrane proteins were studied for a period of 14 weeks, in order to determine whether the red cell metabolism status would support further studies aiming at increasing red cell preservation and viability for blood bank purposes.

In this present work biochemical evaluation of RBC during preservation in ADSOL, for up to 14 weeks at 4°C, is reported. Red blood cell ATP, 2,3-DPG levels, glycolytic kinase and selected dehydrogenase activities and membrane protein fractions were made throughout the 14 weeks.

## METHODS

The procedures that follow were in accordance with the ethical standards of the committee responsible for human experimentation and with the Helsinki Declaration of 1975, as revised in 1983.

Venous blood units of 450 ml were collected in experimental quadruple Blood-Packs^®^ with a primary container having CPDA-1 and one of the satellite bags containing ADSOL (Fenwal code 4R1412, Travenol Laboratories, Inc.). Withdrawals of 450 ml of blood were made from each of 6 healthy adults of both sexes, with ages ranging from 22 to 49 years.

The blood units were centrifuged at 600 g at 4°C for 30 minutes. The supernatant plasma was transferred to the first satellite pack, and the buffer coating to the second one. The erythrocytes from the first container were resuspended in the same volume of ADSOL (2 mM adenine, 122 mM glucose, 154 mM sodium chloride, and 42 mM mannitol).^2^ The hematocrit suspension was adjusted to 40 to 50%, when necessary. After gentle mixing for at least 20 min, the erythrocyte suspension was transferred to sterile PVC vials and kept at 4°C.

All enzyme assays were performed on the day following collection, at weekly intervals up to 6 weeks, and biweekly thereafter.

Blood pH and extracellular sodium and potassium analyses were carried out in Blood Gaz Analyzer (Instrumentation Laboratory, Inc.) at Universidade Federal do Paraná, Curitiba. Assays of erythrocyte adenosine-5’-triphosphate (ATP), 2,3-diphosphogly- cerate (2,3-DPG) concentrations, and hexokinase (Hx), phosphofructokinase (PFK) pyruvate kinase (PK), glyceraldehyde-3-phosphate dehydrogenase (GAPD), glucose-6-phosphate dehydrogenase (G6-PD), 6-phosphogluconate dehydrogenase (6-PGD), glutathione reductase (GR) and glutathione peroxidase (GSHPx) activities were made according to standard methods^10^ in a Gilford spectrophotometer 2451, in the LIM-23 of the Psychiatry Institute of the Hospital das Clínicas of the Faculty of Medicine of the University of São Paulo.

Red cell membrane proteins prepared according to Dodge et al.^11^ were applied in sodium dodecyl sulfate - polyacrylamide gel electrophoresis (SDS-PAGE). The Lowry method^12^ was employed for protein assay. SDS-PAGE was performed according to Laemmli.^13^

## RESULTS

**Figure 1:**
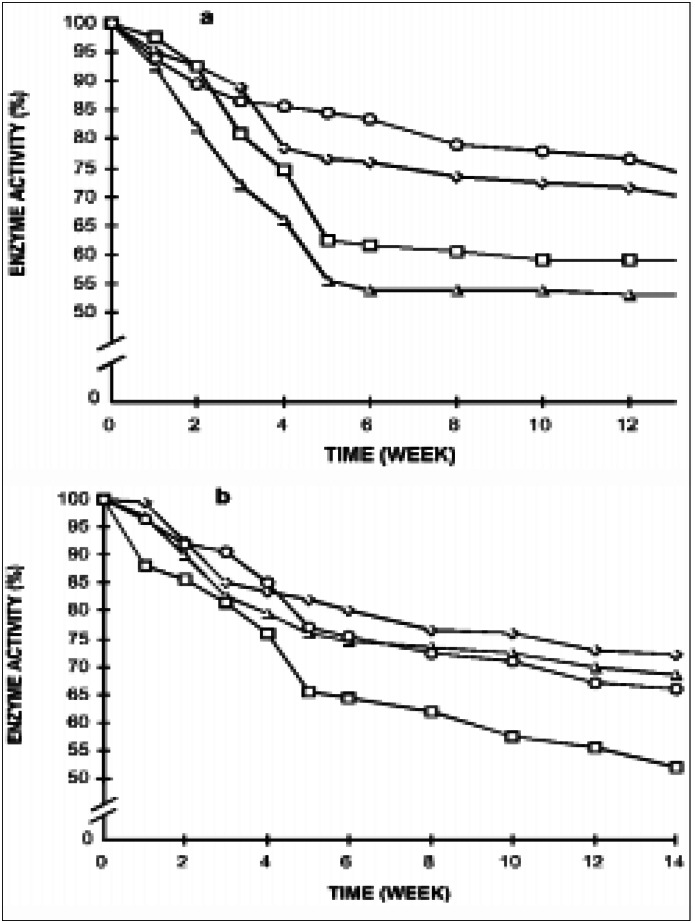
Erythrocyte enzymes activities during red blood cell preservation in ADSOL. a) Ο - G6PD; ◊ - Hx; • - PFK; Δ - PK Initial activity: G6PD - 11.33 IU; Hx - 1.14 IU; PFK - 12.42 IU; PK -13.88 IU; b) ◊ - 6PGD; Δ - GAPD; Ο - GR; • - GSHPX; Initial activity: 6PGD - 8.77 IU; GAPD - 292.4 IU; GR - 8.77 IU; GSHPx -43.85 IU.

Changes in pH, ATP and 2,3-DPG of RBC during preservation in ADSOL are shown in Table 1, in which a general decrease during blood storage may be observed. The Hx, PFK, PK, GAPD, G6-PD, 6-PGD, GR, and GSHPx activities during red blood cell preservation in ADSOL are shown in Figure 1, in which a variable decrease may be observed during the 14 weeks.

**Table 1:** Extracellular pH, and ATP and 2,3 DPG concentrations during red blood cell preservation in ADSOL. Initial concentrations: ATP – 4.95 mmoles/g hemoglobin; 2,3-DPG – 12.15 mmoles/g hemoglobin

	TIME (weeks)										
	0	1	2	3	4	5	6	8	10	12	14
ATP (%)	100(0)	97(4)	89(6)	77(8)	60(15)	45(6)	40(11)	25(11)	16(1)	10(0)	–
PH	6.9(0)	6.8(0)	6.7(0)	6.6(0)	6.6(0.1)	6.5(0.1)	6.5(0.1)	6.4(0.1)	6.4(0.1)	-(0)	6.3(0)
2,3-DPG (%)	100(0)	98(0)	51(1)	13(1)	7(4)	2(1)	1(0)	–	–	–	–

Sd- Standard deviation in parenthesis. Each experimental values is average of six samples.

**Figure 2:**
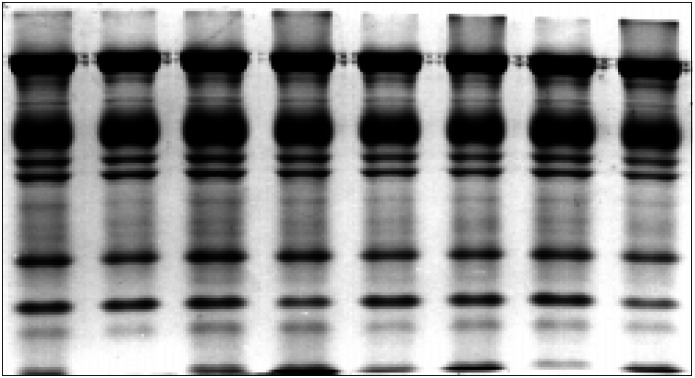
Protein membrane SDS-PAGE of RBC ADSOL preserved RBC. Electrophoresis was carried out according to Laemmli's system, with 10% acrylamide in the running gel and 3% acrylamide in the stacking gel. 100 mg membrane protein were loaded on every well. The runs correspond, from left to right, to 0, 2, 4, 6, 8, 10, 12 and 14 weeks of ADSOL preservation.

**Figure 3:**
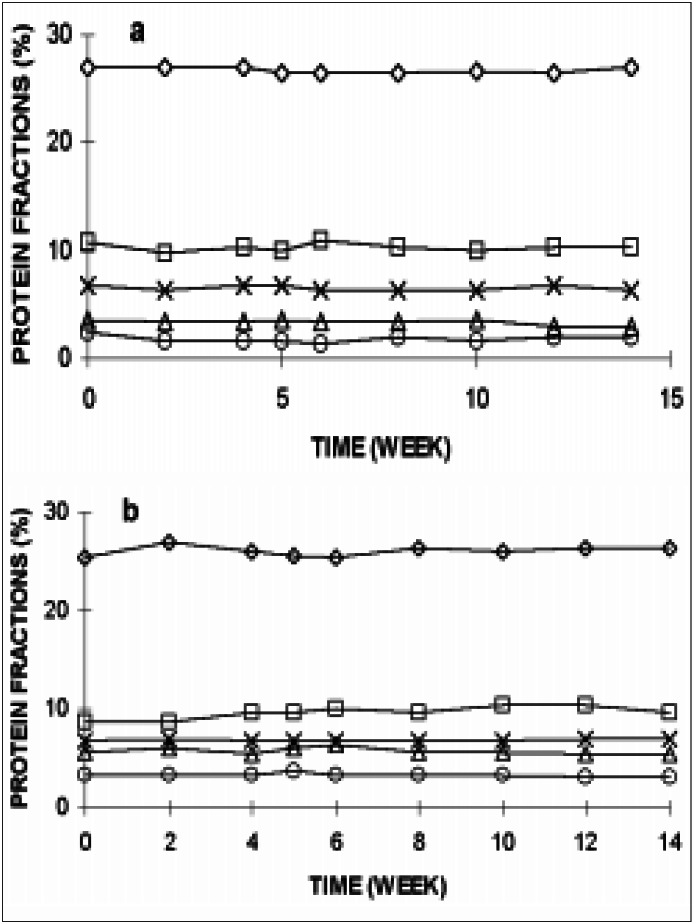
Erythrocyte membrane proteins on SDS-PAGE of ADSOL preserved RBC. a) ◊ - espectrin; Δ - band 2.1; X – band 4.1; o - band 4.9; • - band 5; b) ◊ - band 3; Δ - band 7; X – band 4.2; o - band 4.5; • - band 6

The results of SDS-PAGE RBC membrane protein analysis during ADSOL preservation are shown in Figs. 2 and 3, and no changes were detected during blood storage.

## DISCUSSION

It is well known that during blood preservation with ADSOL, plasma sodium and potassium decrease, red cell adenosine-triphosphate and 2,3-diphosphoglycerate decrease as well, blood hydrogen ion concentration increases, and hemolysis occurs.^14-16^ In this present paper we obtained similar data to other authors regarding ATP, 2,3-DPG and extracellular pH, and other parameters,^2,14-16^ which can be seen in Table 1. However, there are no studies of red cell enzymes or of membrane proteins during longer ADSOL storage periods.

As the glycolytic kinases are involved in the energy generation represented by ATP formation, essential to keep the Na-K pump in activity, all of them were studied. Selected dehydrogenases (from glycolysis, the pentose cycle and related ones), which keep the reduced nicotinamide adenine dinucleotide (NADH) and the reduced nicotinamide adenine dinucleotide phosphate (NADPH) nucleotides in their reduced state, were also investigated. These reduced nucleotides reduce the dangerous peroxides and disulfide bridges, which damage the membrane proteins and other proteins.

Enzyme activity decay was observed during the storage, so that by the 5th week, hexokinase, glyceraldehyde phosphate dehydrogenase, glutathione reductase, glucose-6-phosphate dehydrogenase, and 6-phosphogluconate dehydrogenase fell 30%, phosphofructokinase and glutathione peroxidase 35%, and pyruvate kinase 45% (Figure 1). Thereafter, a common 10% decrease among all enzymes was observed up to the 14th week. As all enzymes lost activity during the first 5 weeks, it hints that those unstable forms of enzymes that depend on fine physiological environmental requirements early become inactive. But, most of the forms do keep their functional properties until the 14th week, suggesting that they represent more stable enzyme forms in spite of the decrease in pH, nucleotides and phosphate compounds.

These data suggest that ADSOL-preserved red cells keep their basic biochemical characteristics throughout the 14 weeks, although presenting early ATP and 2,3-DPG depletion.

Studies in different preservative solutions have reported variable enzymatic activity changes during *in vitro* RBC preservation.^15-17^ Noble et al.^15^ reported 0-20% reduction in activity for several enzymes and 33% for phosphofructokinase in CPDA-1 preserved red cells for up to 5 weeks. Mourad et al.^17^ observed a 25% activity decrease after 7 weeks and 30-50% after 19 weeks of preservation in ACD. Nakao et al.^18^ observed stable hexokinase activity over 8 weeks when ACD preservative solution plus adenine and inosine was used. ATP depletion after 8 weeks of preservation in ACD was recovered after adenine and inosine addition, despite the 50% decrease in hexokinase activity. Although hexokinase is the first glycolytic enzyme, the remaining enzymatic activity seems to keep the functional metabolic pathways.

Red cell membrane proteins during storage have been studied under different conditions.^19-22^ Some re-ports^21,22^ describe high MW oligomer formation, which was ascribed to membrane protein interactions. Wolfe et al.^19^ described a decrease in the spectrin-actin interaction, no oligomer formation and membraneglobin association in CPD, while Schrier et al.^21^ described oligomer and actin increase, as well as band 2, 3, 4.1 and 4.2 decrease during storage in CPD-A2.

Significant membrane protein changes could represent a RBC life-limiting storage lesion.^23^ Therefore we performed the membrane protein SDS-PAGE analysis in order to detect any modification during the 14 weeks. No significant change in SDS-PAGE during ADSOL preservation was observed, as can be observed in Figures 2 and 3. No oligomers were formed during the 14 weeks in ADSOL-preserved red cells, whereas it occurs when CPD and CPDA-2 are used.^20-22^ It is possible that this difference may be ascribed to the technical procedure or to the preservative solutions employed by the other authors. According to our data, ADSOL seems to be a better preservative solution regarding oligomer generation and relative concentration of membrane proteins.

Although the ADSOL-preserved red cells keep their basic metabolic functions and membrane structure for up to 14 weeks, they are not adequate for blood transfusion, as ATP and 2,3-DPG decrease a great deal. However, the fairly good enzymes conditions up to 14 weeks are encouraging for making greater efforts towards trying to restore red cell ATP and 2,3-DPG levels for longer, enhancing blood viability.

The ADSOL-preserved red cell enzymes present a standard and gradual activity decay. Thus, if G-6-PD is assayed in red cells preserved in ADSOL for 8 or 12 weeks, the observed activity will represent 60% of the initial values (-30% after 5 weeks plus -10% by the 8th to 12th week), and a reliable value may be given. Moreover, red cells in which the enzyme activities are assayed as soon as blood is collected may be used as standards, and blood samples may be sent to other laboratories, which will have them as standards. The same may be extended for membrane proteins, which are very stable with ADSOL for at least 14 weeks.
